# Editorial: Recent advances in thromboembolism and oral contraceptives

**DOI:** 10.3389/fendo.2026.1812743

**Published:** 2026-03-10

**Authors:** L. Morimont, J. M. Foidart

**Affiliations:** 1 Research Unit in Clinical Pharmacology and Toxicology (URPC), NAmur Research Institute for LIfe Sciences (NARILIS), Faculty of Medicine, University of Namur, Namur, Belgium; 2 Department of Obstetrics and Gynecology, University of Liege, Liege, Belgium

**Keywords:** combined oral contraceptive (COC), estetrol (E4), estradiol, ethinylestradiol, venous thromboembolism (VTE)

Combined oral contraceptives (COCs) remain among the most widely used reversible methods of contraception worldwide. Given the substantial number of thrombosis cases associated with their use—approximately 22,000 annually in the European Union (1) —it is essential for healthcare professionals to identify women at increased risk before prescribing COCs. This Research Topic seeks to bring renewed clarity to how different estrogen–progestin combinations influence venous thromboembolism (VTE) risk. Beyond simply comparing formulations, it invites a broader reflection on the pharmacokinetic and pharmacodynamic properties of these hormones and how they shape the coagulation response. Ultimately, it raises an important question for the future of women’s health: could dedicated screening tools or biomarkers help identify women at higher thrombotic risk before initiating COCs, and thereby guide safer contraceptive choices?

Historically, COC formulations relied almost exclusively on synthetic estrogens—most commonly ethinyl estradiol (EE)—combined with various progestins. In recent years, however, the introduction of body identical estrogens, such as estradiol (E2) (and its valerate form) and estetrol (E4), have reshaped the landscape of COC development and reopened the discussion on estrogen choice. Comparative evaluations of estrogenic components used in hormonal contraception indicate that natural estrogens, particularly E4, offer notable advantages in terms of metabolic safety and tolerability, while exerting a reduced hepatic impact and a lower associated risk of VTE (2). Within this Research Topic, the review and meta-analysis by Douxfils et al. Presents a compelling comparison between natural and synthetic estrogens, consistently demonstrating a lower VTE risk with natural estrogen–containing COCs. Adding real-world depth to these findings, Didembourg et al. (3) performed a disproportionality analysis using the EudraVigilance database to capture actual VTE cases reported following COC use. Their analysis—reflecting what truly occurs in routine clinical practice—showed markedly lower reporting rates for natural estrogen formulations compared with EE–levonorgestrel combinations: 0.24 (95% CI 0.17–0.33) for E4/drospirenone, 0.44 (95% CI 0.38–0.51) for E2/nomegestrol acetate, and 0.45 (95% CI 0.41–0.49) for E2/dienogest. Complementing these clinical and real-world insights, Stanczyk et al. Provide mechanistic context, detailing how different estrogens modulate coagulation pathways and helping to explain the divergence in VTE risk. Together, these contributions offer clinical evidence and biological rationale needed to rethink estrogen selection in modern contraception.

This perspective aligns seamlessly with the work of the Estrogen and Combined Oral Contraceptive Expert Committee (ESCONEC) Project, presented at the International Federation of Gynecology and Obstetrics (FIGO) World Congress in Cape Town, South Africa (October 2025). The ESCONEC aims to articulate a clear rationale for shifting toward natural estrogens in COCs. By more closely reflecting the physiology of natural estrogens, E2(V) and E4 reduce hepatic burden and support a more favorable balance across coagulation, lipid, and bone health markers. Yet current contraceptive guidelines have not fully integrated the increasing body of evidence supporting their use. The project seeks to bridge this gap and stimulate a renewed, evidence-based dialogue on estrogen choice in women’s health (4).

The choice of progestin also plays a pivotal role. As highlighted by Gaspard et al., progestins differ markedly in their pharmacokinetics, metabolic pathways, and receptor-binding profiles, confirming the absence of a class effect. In the context of EE-containing COCs, EE/levonorgestrel has consistently been associated with the lowest VTE risk. This apparent advantage, however, is largely driven by the androgenic properties of levonorgestrel, which partially counteract EE-induced increases in SHBG and hepatic protein synthesis. By contrast, combinations such as EE/desogestrel or EE/drospirenone—which involve less androgenic or even anti-androgenic progestins—allow the full hepatic impact of EE to be expressed, resulting in a higher thrombotic risk. Importantly, this does not reflect a genuinely favorable hemostatic profile for levonorgestrel itself, but rather a relative mitigation within an intrinsically high-risk EE environment.

A different picture emerges when EE is replaced by natural estrogens such as E2(V) or E4. In this context, the properties of the progestin become essential in maintaining the inherently lower hepatic impact of these hormones. Non-androgenic or anti-androgenic progestins such as nomegestrol acetate, dienogest and drospirenone are particularly well suited to body identical estrogens. These progestins exhibit no affinity for SHBG and exert minimal hepatic stimulation, thereby avoiding the amplification of coagulation factors typically driven by EE. Their neutral—or even favorable—hemostatic profiles allow the intrinsic advantages of body identical estrogens to be fully expressed, including reduced SHBG induction, lower activated protein C(APC) resistance, and attenuated thrombin generation (5). As a result, combinations such as E2/nomegestrol acetate, E2V/dienogest, or E4/drospirenone create a more physiological endocrine environment and consistently demonstrate lower thrombotic signals across clinical studies, real-world pharmacovigilance, and mechanistic hemostasis data.

Finally, the cost-effectiveness analysis conducted by Douxfils adds an important societal dimension. His findings show that implementing a screening strategy prior to COC initiation could not only prevent thrombotic events but also yield substantial economic benefits for healthcare systems. It is estimated that replacing EE with E2 or E4 in COCs could prevent between 7,000 and 10,000 VTE cases annually in the European Union, potentially saving 400 to 600 lives per year. This evidence underscores the broader public health value of moving toward more individualized, and ultimately safer, contraceptive prescribing.

Together, the evidence presented in this Research Topic converges toward a simple yet compelling message: estrogen choice matters. Body identical estrogens, when associated with non-androgenic or anti-androgenic progestins, offer a more physiological and ultimately safer alternative to conventional EE-based COCs, with reduced hepatic stimulation. They show that elevated thrombotic risk should no longer be viewed as an unavoidable consequence of COC use.

As clinical, mechanistic, real-world, and economic data now align in favor of these newer combinations, the challenge ahead lies not in generating further evidence, but in translating it into practice. Updating contraceptive guidelines, developing individualized screening strategies, and ensuring equitable access to safer formulations will be essential steps in this evolution. The shift toward body identical estrogen–based COCs is no longer a theoretical proposition — it is an evidence-driven opportunity to improve women’s health on a global scale.
